# CHALLENGES IN RECEIVING, ORGANIZING, AND PROVIDING CARE IN ACTIVITIES OF DAILY LIVING: A WORLD-CAFé DIALOGUE

**DOI:** 10.1093/geroni/igz038.1830

**Published:** 2019-11-08

**Authors:** Svenja Cremer, Michel Bleijlevens, Silke F Metzelthin, Janneke M de Man-van Ginkel, Sandra M Zwakhalen

**Affiliations:** 1 Department of Health Services Research, Care and Public Health Research Institute (CAPHRI), Maastricht University, Maastricht, The Netherlands, Netherlands; 2 Department of Health Services Research, Care and Public Health Research Institute (CAPHRI), Maastricht University., Limburg, Netherlands; 3 Maastricht University, Department of Health Services Research, Care and Public Health Research Institute (CAPHRI), Maastricht, Netherlands; 4 University Medical Center Utrecht, Utrecht, Netherlands; 5 Maastricht University, School for Public Health and Primary Care (CAPHRI), Department of Health Services Research, Living Lab in Ageing and Long-term Care, Maastricht, Limburg, Netherlands

## Abstract

Activities of Daily Living are a series of basic activities performed by individuals on a daily basis necessary for independent living at home or in the community. ADL are part of basic nursing care which describe aspects of care that are fundamental to all patients’ health and well-being, regardless of diagnosis, cultural background or healthcare setting. In daily practice, nursing staff is confronted with challenges when providing ADL-care. In order to provide nursing professionals with practical tools to tackle daily challenges, a deeper insight into the perceived challenges by nursing staff, care receivers and informal caregivers is necessary. Therefore, two ‘world-café’ sessions were organized to explore daily challenges in ADL-care. In a World Café, participants are regarded as experts of their own lived experience and experiential knowledge. By creating a hospitable environment (i.e. a café-style ambience) individual and collective knowledge and ideas can be shared. Care receivers, informal caregivers and professionals including nurses, nursing-assistants, physical therapists, occupational therapist, and other stakeholders (N=53) participated in these ‘world-cafés’. The session’s output comprised ‘graphic recordings’ (paper tablecloths and post its) of the conversations that have been analyzed using content analysis. Findings showed reported challenges regarding receiving, providing and organizing ADL-care. Challenges on organizational level comprised for example both bilateral agreements and communication. Whereas reported care related issues related to challenges such as the assessment of care receivers’ needs and the use of assistive devices in the provision of ADL-care. The inventoried challenges will be addressed in an ADL-quality standard for nursing staff.

